# Third-Trimester NT-proBNP for Pre-eclampsia Risk Prediction: A Comparison With sFlt-1/PlGF in a Population-Based Cohort

**DOI:** 10.1016/j.jacadv.2025.101671

**Published:** 2025-03-19

**Authors:** Lucas Bacmeister, Annette Buellesbach, Dorte Glintborg, Jan Stener Jorgensen, Birgitte Møller Luef, Anna Birukov, Adrian Heidenreich, Diana Lindner, Till Keller, Kristin Kraeker, Tanja Zeller, Ralf Dechend, Marianne Skovsager Andersen, Dirk Westermann

**Affiliations:** a Clinic for Cardiology and Angiology, University Heart Center Freiburg – Bad Krozingen, Medical Center – University of Freiburg, Faculty of Medicine, University of Freiburg, Freiburg, Germany; b Department of Endocrinology, Odense University Hospital, University of Southern Denmark, Odense, Denmark; c Institute for Clinical Research, Faculty of Health Sciences, University of Southern Denmark, Odense, Denmark; d Department of Obstetrics and Fetal Medicine, Odense University Hospital, University of Southern Denmark, Odense, Denmark; e Department of Nutrition, Harvard T.H. Chan School of Public Health, Boston, Massachusetts, USA; f Experimental and Clinical Research Center, A Cooperation Between the Max-Delbrück-Center for Molecular Medicine in the Helmholtz Association and the Charité - Universitätsmedizin Berlin, Berlin, Germany; g Department of Cardiology, University Heart and Vascular Center Hamburg, Medical University Hamburg-Eppendorf, Hamburg, Germany; h Institute for Cardiogenetics, University of Luebeck, University Hospital Schleswig-Holstein, Luebeck, Germany; i German Center for Cardiovascular Research, DZHK, Partner Site Hamburg/Kiel/Lübeck, Hamburg, Germany; j Department of Cardiology and Nephrology, HELIOS Clinic, Berlin, Germany

**Keywords:** cardio-obstetrics, hypertension, natriruretic peptides, pre-eclampsia, risk prediction

## Abstract

**Background:**

The association between lower first-trimester N-terminal pro B-type natriuretic peptide (NT-proBNP) levels and increased pre-eclampsia risk remains poorly understood, contrasting with the elevated NT-proBNP levels observed at the time of pre-eclampsia diagnosis.

**Objectives:**

The aim of this study was to assess the utility of third-trimester NT-proBNP for assessing pre-eclampsia risk before onset.

**Methods:**

NT-proBNP and the soluble Fms-like tyrosine kinase 1 to placental growth factor ratio (sFlt-1/PlGF) were measured in 1,476 pregnant individuals from the Odense Child Cohort at a median gestational age of 29 weeks (Q1-Q3: 28.4-29.4). Pre-eclampsia cases were categorized by timing: 11 individuals (0.7%) developed pre-eclampsia within 4 weeks, while 110 (7.5%) developed pre-eclampsia more than 4 weeks after sampling.

**Results:**

Higher NT-proBNP levels were significantly associated with increased risk of pre-eclampsia within 4 weeks but reduced risk beyond 4 weeks. After adjusting for age, body mass index, nulliparity, systolic blood pressure, and the sFlt-1/PlGF ratio, the adjusted OR was 2.18 (95% CI: 0.88-5.42, *P* = 0.09) for onset within 4 weeks and 0.72 (95% CI: 0.55-0.93, *P* = 0.012) for onset beyond 4 weeks. However, combining NT-proBNP with the sFlt-1/PlGF ratio did not improve the predictive accuracy for short- or long-term pre-eclampsia risk compared to the sFlt-1/PlGF ratio alone.

**Conclusions:**

Unselected NT-proBNP screening in the early third trimester has limited clinical value for predicting short- or long-term pre-eclampsia risk when compared to angiogenic biomarkers.

Pre-eclampsia causes significant maternal and fetal morbidity and is associated with postpartum cardiovascular disease.^1^, ^2^, ^3^, ^4^ Vice versa, this syndrome, especially when manifesting preterm, shares numerous risk factors with cardiovascular diseases.^5^^,^^6^ Natriuretic peptides, such as brain natriuretic peptide (BNP) and its inert byproduct N-terminal pro B-type natriuretic peptide (NT-proBNP), are established markers for cardiac congestion and guide heart failure management outside of pregnancy.^7^

During pregnancy, the value of angiogenetic markers, particularly the ratio of soluble Fms-like tyrosine kinase 1 to placental growth factor (sFlt-1/PlGF), is well established for pre-eclampsia prediction, effectively identifying individuals at risk within 4 weeks of assessment.^8^ Recently, our group has demonstrated that high-sensitivity cardiac troponin I enhances pre-eclampsia prediction.^9^ Elevated natriuretic peptides have been reported at the time of pre-eclampsia diagnosis, especially when the disease manifests preterm.^10^, ^11^, ^12^ Conversely, an inverse relationship between first-trimester NT-proBNP levels and the risk of pre-eclampsia has been observed.^13^

This reversal of the direction of the association between natriuretic peptide levels and pre-eclampsia risk across gestation remains poorly understood. In nonpregnant individuals, NT-proBNP levels increase weeks before the onset of cardiovascular events.^14^ Subclinical cardiac pathologies, such as diastolic dysfunction, often precede the manifestation of pre-eclampsia.^15^, ^16^, ^17^, ^18^ This suggests that natriuretic peptides could have analogous value when measured in temporal proximity to pre-eclampsia onset.

We hypothesized that elevated NT-proBNP levels in the early third trimester may precede the clinical manifestation of pre-eclampsia and provide additional value for short-term prediction within the 4-week window validated for sFlt-1/PlGF.

## Methods

### Study design

#### Odense child cohort

The Odense Child Cohort is a population-based cohort study conducted in the Municipality of Odense, Denmark, focusing on pregnant women and their newborns from January 1, 2010, to December 31, 2012.^19^ All pregnant women residing in the Odense municipality during this time were eligible to participate. A total of 2,874 women, representing 43% of the registered pregnancies during the study period, were enrolled. Detailed aims, design, and cohort profiles have been outlined previously.^19^

## Study population

**Figure 1:**
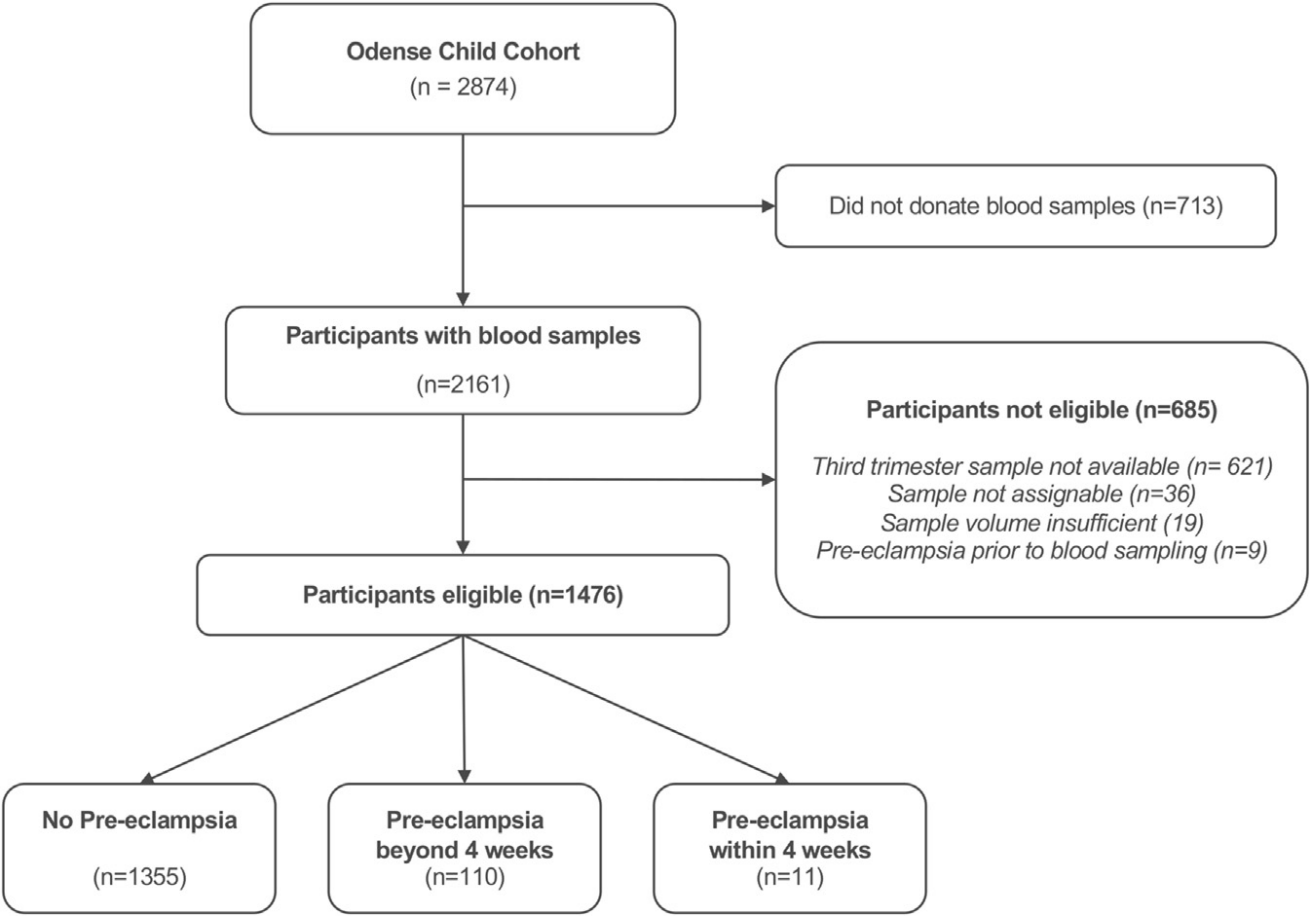
**Flow Chart of Study Eligibility** Two thousand eight hundred seventy-four individuals participated in the Odense Child Cohort. Blood samples were available for 2,161 individuals. A total of 1,476 participants were eligible for study inclusion, of whom 110 individuals developed pre-eclampsia beyond 4 weeks after blood sampling, whereas 11 individuals developed the disease within 4 weeks.

Out of the total cohort, 2,161 participants provided blood samples at 10 and/or 28 weeks of gestation (Figure 1). Of these, 685 participants were not eligible for further analysis: 621 did not provide a third-trimester sample, 36 aliquots were unassignable, and 19 had insufficient sample volume. Additionally, 9 participants had a pre-eclampsia diagnosis prior to sampling, leading to a final study population of 1,476 individuals with third-trimester blood samples available for biomarker analysis (Figure 1).

In the comparison between participants eligible for study inclusion (N = 1,476) and those not eligible (N = 685), there were no significant differences in maternal age, body mass index (BMI), incidence of pre-eclampsia, twin pregnancies, first trimester systolic blood pressure, hypertension, diabetes, chronic kidney disease, birth weight, placental weight, or newborn sex (Supplemental Table 1). However, significant differences were observed for nulliparity (60.0% in eligible participants vs 49.2% in noneligible participants, *P* < 0.001) and smoking rates (3.7% vs 7.2%, *P* < 0.001).

### Definition of pre-eclampsia

Pre-eclampsia was defined as blood pressure ≥140/90 mm Hg with proteinuria (>0.3 g/d or at least +1 on sterile urine dipstick) after 20 + 0 weeks of gestation.^20^ The diagnoses of pre-eclampsia were validated retrospectively by evaluation of the electronic patient files of all included women.^21^ Pre-eclampsia cases were categorized based on the time between blood sampling and diagnosis: onset within 4 weeks and onset beyond 4 weeks. For supplementary analyses, pre-eclampsia cases were further classified by gestational age at onset: early-onset (at or before 34 + 0 weeks), late-onset preterm (before 37 + 0 weeks), and late-onset at term (at or after 37 weeks).

### Measurement of NT-proBNP and sFlt-1/PlGF

Serum samples were collected via venous blood draw, promptly centrifuged, and stored in aliquots at −80 °C in the institutional biobank until analysis.^19^

### NT-proBNP

NT-proBNP was determined centrally in a single batch using a commercially available assay (Alere NT-proBNP for Architect, Abbott) on an ARCHITECT i2000 system, with a limit of detection of 4.9 pg/mL. Among the 1,476 samples successfully analyzed, 98.5% (1,454 aliquots) had NT-proBNP levels ≥5.0 pg/mL. For samples with values below this threshold (n = 22), a value of 5.0 pg/mL was assigned. No sample had a value above the upper limit of detection (35,000 pg/mL).

### sFlt-1 to PlGF ratio

sFlt-1 and PlGF levels were measured in a single batch using the fully automated KRYPTOR compact PLUS system with commercially available immunoassays (KRYPTOR PlGF and KRYPTOR sFlt-1; Thermo Fisher Scientific), as previously described.^22^ The detection limits for PlGF and sFlt-1 were 3.6 pg/mL and 22 pg/mL, respectively, while their upper limits were 7,000 pg/mL for PlGF and 90,000 pg/mL for sFlt-1.^23^ No sample had values outside these boundaries.

### Statistical analysis

Continuous variables are presented as median with 25th to 75th percentile (Q1-Q3), while categorical variables are expressed as counts and percentages. Group comparisons were made using the Mann-Whitney test for continuous variables and Fisher exact test for categorical variables. When comparing more than 2 groups, the Kruskal-Wallis test was used for continuous variables and the chi-square test for categorical variables.

Kaplan-Meier probability curves were constructed to visualize time to pre-eclampsia onset since blood sampling. Time-to-event was calculated from the date of blood sampling to the diagnosis of pre-eclampsia. Censoring was applied at 100 days of follow-up or at delivery for individuals who did not develop pre-eclampsia. Groups were stratified by NT-proBNP ≥50 pg/mL. Given that the association between NT-proBNP and pre-eclampsia changed over time, proportional hazards assumptions were not met. Hence, multinomial logistic regression to account for the time-dependent association between NT-proBNP and pre-eclampsia onset was employed. ORs for pre-eclampsia onset within 4 weeks and beyond 4 weeks were estimated within the same multinomial logistic regression model, using no pre-eclampsia as the reference category. Regression analyses were both conducted in crude form and adjusted for maternal age, BMI, nulliparity, systolic blood pressure at the time of sampling, and the sFlt-1/PlGF ratio. Due to non-normal distribution, NT-proBNP, the sFlt-1/PlGF ratio, and maternal BMI were log-transformed before using them in regression analyses. ORs for a one-unit change on the log scale, along with their respective 95% CIs, are presented.

Receiver operating characteristic (ROC) curves were applied to assess the predictive capacity of NT-proBNP, sFlt-1/PlGF, and their combination for predicting pre-eclampsia within 4 weeks, beyond 4 weeks, and the composite of all pre-eclampsia cases. Area under the ROC curves (AUCs), along with their respective 95% CIs, are presented.

All statistical analyses were conducted using R version 4.3.1 (R Foundation for Statistical Computing.

## Results

### Cohort characteristics by time to pre-eclampsia onset

Of 1,476 individuals included in this study, 121 (8.2%) developed pre-eclampsia after blood sampling, as shown in Table 1. Of these, 11 individuals (0.7%) developed pre-eclampsia within 4 weeks, while 110 (7.5%) developed pre-eclampsia beyond 4 weeks. The median time to pre-eclampsia onset was 14.0 days (Q1-Q3: 10.0-24.5 days) for those developing it within 4 weeks and 65.5 days (Q1-Q3: 52.0-77.8 days) for those developing it later (*P* < 0.001). The median gestational week at blood sampling for the entire cohort was 29.0 (Q1-Q3: 28.4-29.4), with no significant difference between the groups (*P* = 0.594).

**Table 1:** Baseline Characteristics of the Study Cohort Stratified by Pre-eclampsia Occurrence Within or Beyond 4 Weeks

	No Pre-eclampsia (n = 1,355)	Pre-eclampsia Beyond 4 Wk (n = 110)	Pre-eclampsia Within 4 Wk (n = 11)	*P* Value
Maternal age (y)	30.0 (27.0, 33.0)	31.0 (27.0, 34.0)	33.0 (27.0, 37.0)	0.41
BMI (kg/m^2^)	23.4 (21.3, 26.2)	25.4 (22.4, 28.7)	28.3 (24.4, 36.5)	**<0.001**
Nulliparity	789 (58.2%)	85 (77.3%)	11 (100%)	**<0.001**
Gestational age at blood sampling (wk)	29.0 (28.4, 29.4)	29.0 (28.4, 29.4)	28.6 (28.4, 29.1)	0.59
Time to pre-eclampsia manifestation (d)	-	65.5 (52.0, 77.8)	14.0 (10.0, 24.5)	**<0.001**
Gestational age at delivery (wk)	40.1 (39.1, 41.0)	39.8 (38.3, 40.5)	37.3 (34.9, 38.2)	**<0.001**
SBP 1st trimester (mm Hg)	115 (110, 122) (*missing: n = 139*)	120 (114, 130) (*missing: n = 14*)	128 (121, 142) (*missing: n = 1*)	**<0.001**
SBP at sampling (mm Hg)	117 (110, 125) (*missing: n = 54*)	126 (119, 133) (*missing: n = 3*)	146 (136, 150) (*missing: n = 1*)	**<0.001**
Hypertension	50 (3.7%)	7 (6.4%)	1 (9.1%)	0.29
Diabetes	7 (0.5%)	1 (0.9%)	0 (0%)	0.84
Kidney disease	3 (0.2%)	1 (0.9%)	0 (0%)	0.40
Birth weight (g)	3,520 (3,180, 3,860)	3,370 (2,970, 3,770)	2,270 (1,790, 2,960)	**<0.001**
Placental weight (g)	620 (530, 720)	600 (520, 708)	460 (380, 585)	**0.030**
Female offspring	640 (47.2%)	52 (47.3%)	6 (54.5%)	0.89
NT-proBNP (pg/mL)	47.9 (27.9, 79.4)	39.4 (19.8, 54.7)	94.8 (53.3, 165)	**<0.001**
sFlt-1/PlGF	3.13 (1.88, 5.19)	5.23 (3.21, 11.7)	114 (18.4, 166)	**<0.001**

Values are median (Q1, Q3) or n (%). Baseline characteristics of the study participants stratified by pre-eclampsia manifestation within or beyond 4 weeks after blood sampling. *P* values were determined using the Kruskal-Wallis test for continuous variables and the chi-square test for binary variables. **Bold** values indicate *P* values <0.05.

BMI = body mass index; NT-proBNP = N-terminal pro B-type natriuretic peptide; SBP = systolic blood pressure; sFlt-1/PlGF = soluble fms-like tyrosine kinase-1 to placental growth factor ratio.

Maternal BMI, systolic blood pressure at the first trimester, systolic blood pressure at the time of sampling, the sFlt-1/PlGF ratio, twin pregnancy, and nulliparity all showed a stepwise increase from those without pre-eclampsia to those who developed pre-eclampsia more than 4 weeks after sampling to those who developed it within 4 weeks (Table 1). Conversely, gestational age at delivery, birth weight, and placental weight followed a stepwise decrease, with the highest values in those without pre-eclampsia, lower in those developing pre-eclampsia beyond 4 weeks, and lowest in those developing it within 4 weeks.

No significant differences were observed between these groups for maternal age, pre-existing hypertension, pre-existing diabetes, chronic kidney disease, smoking status, or weight gain up to the time of blood sampling.

Cohort characteristics stratified by gestational age at the onset of pre-eclampsia (early-onset, late-onset preterm, and late-onset at term) are provided in Supplemental Table 2.

### NT-proBNP levels by time to pre-eclampsia onset

**Figure 2:**
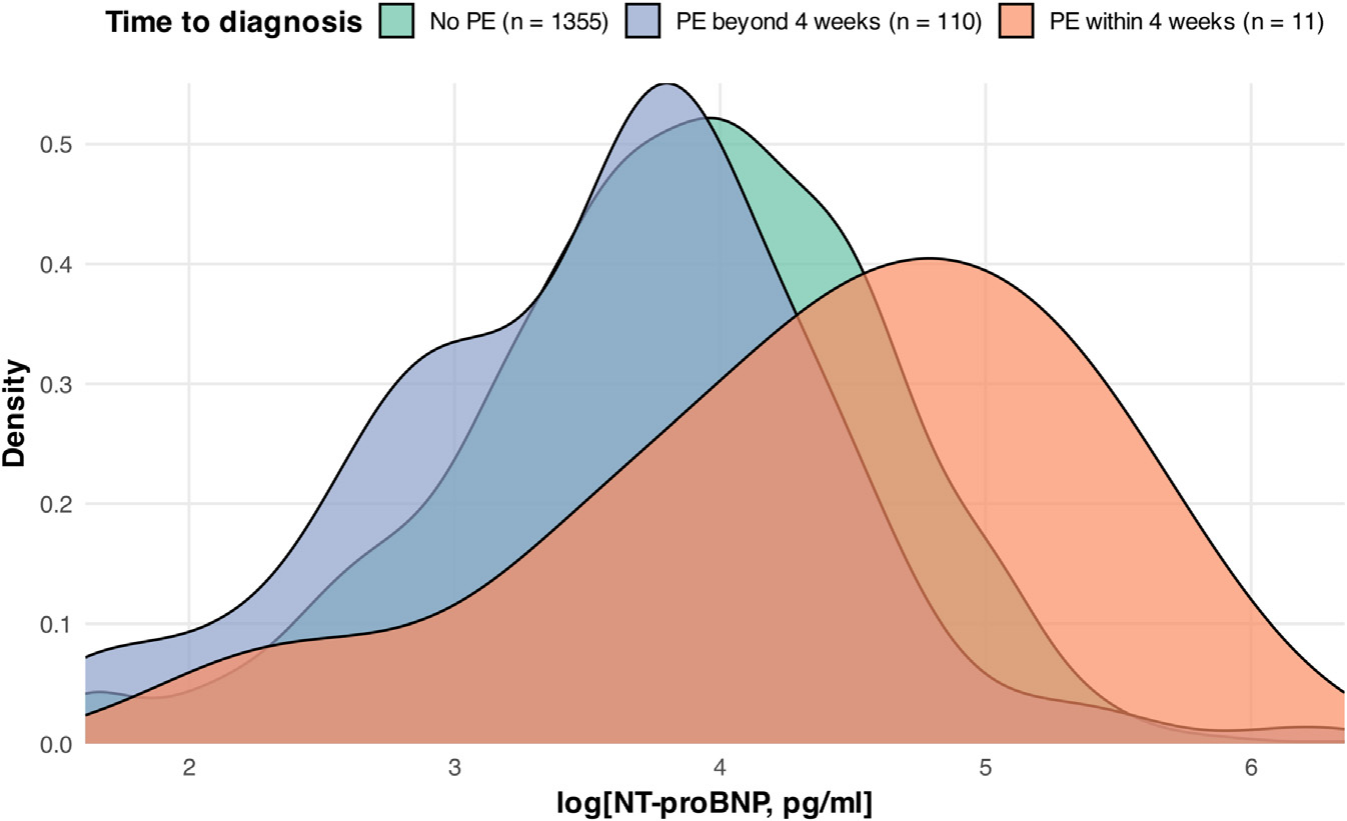
**Density Plot of N-Terminal Pro B-Type Natriuretic Peptide Stratified by Time to Pre-eclampsia Diagnosis** Log-transformed NT-proBNP levels show a right-skewed distribution in individuals who develop pre-eclampsia (PE) within 4 weeks compared to those without disease manifestation. In contrast, individuals who develop the disease beyond 4 weeks exhibit a slight left-skewed distribution compared to those without disease manifestation. NT-proBNP = N-terminal pro B-type natriuretic peptide.

NT-proBNP levels showed significant variation across the groups as shown in Table 1 and visually depicted in the density plot in Figure 2. Individuals who developed pre-eclampsia more than 4 weeks after sampling had lower NT-proBNP levels (median 39.4 pg/mL; Q1-Q3: 19.8-54.7 pg/mL) compared to those without pre-eclampsia during their pregnancy (median 47.9 pg/mL; Q1-Q3: 27.9-79.4 pg/mL). In contrast, individuals who developed pre-eclampsia within 4 weeks had higher NT-proBNP levels (median 94.8 pg/mL; Q1-Q3: 53.3-165.0 pg/mL).

### Associations of NT-proBNP levels and pre-eclampsia risk

**Figure 3:**
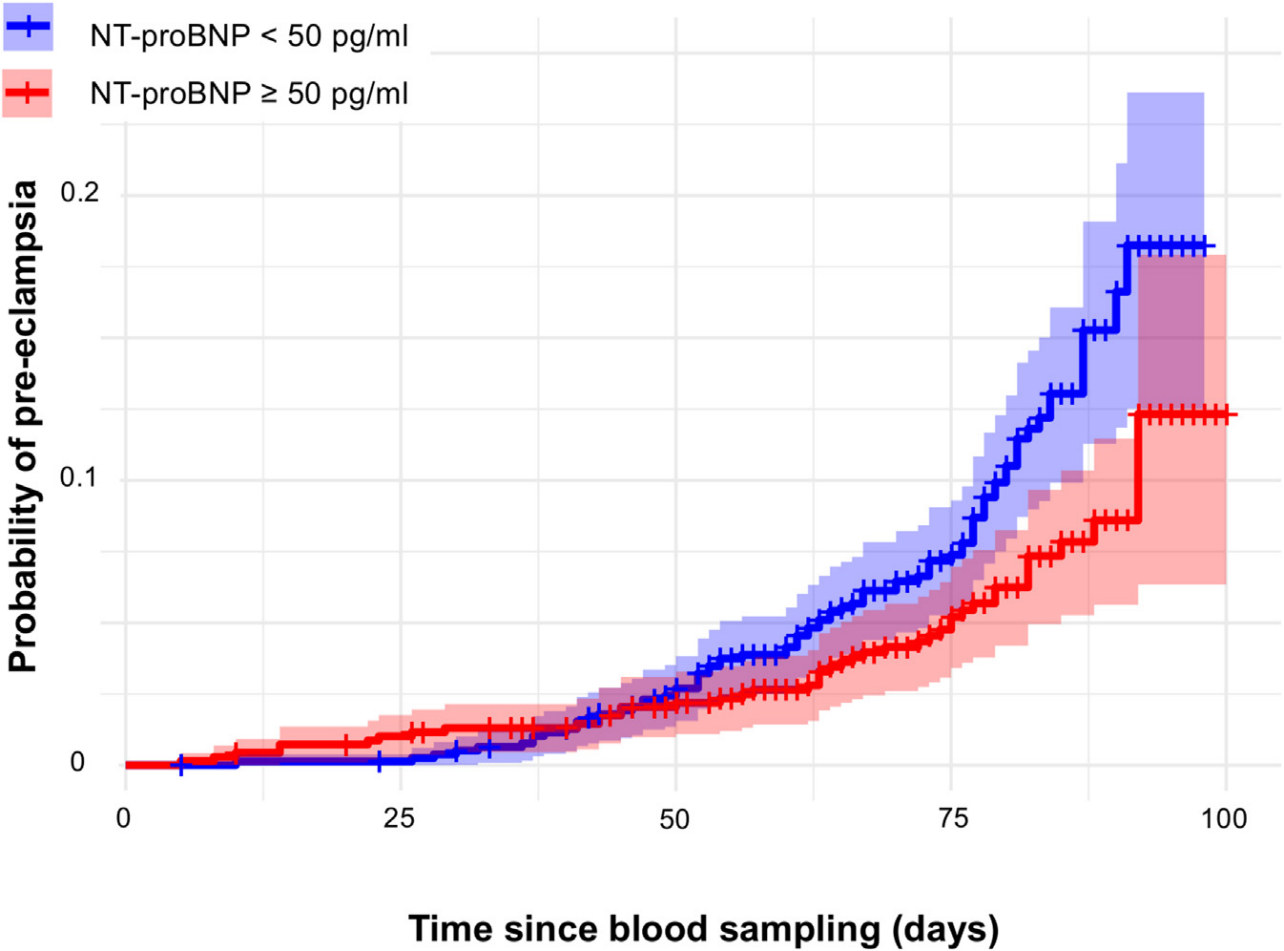
**Probability of Pre-eclampsia Development Stratified by N-Terminal Pro B-Type Natriuretic Peptide ≥50 pg/mL** The probability for pre-eclampsia for individuals with NT-proBNP levels ≥50 pg/mL reversed over the observation period. As proportional hazard assumptions were not met, Cox proportional hazard analysis was not performed. NT-proBNP = N-terminal pro B-type natriuretic peptide.

Kaplan-Meier probability curves visualizing the time-to-pre-eclampsia onset following blood sampling showed that the association between NT-proBNP levels and pre-eclampsia risk reversed over the observation period: initially, individuals with NT-proBNP ≥50 pg/mL had a higher probability of pre-eclampsia, but as gestation advanced, their risk became lower compared to those with NT-proBNP <50 pg/mL (Figure 3).

**Figure 4:**
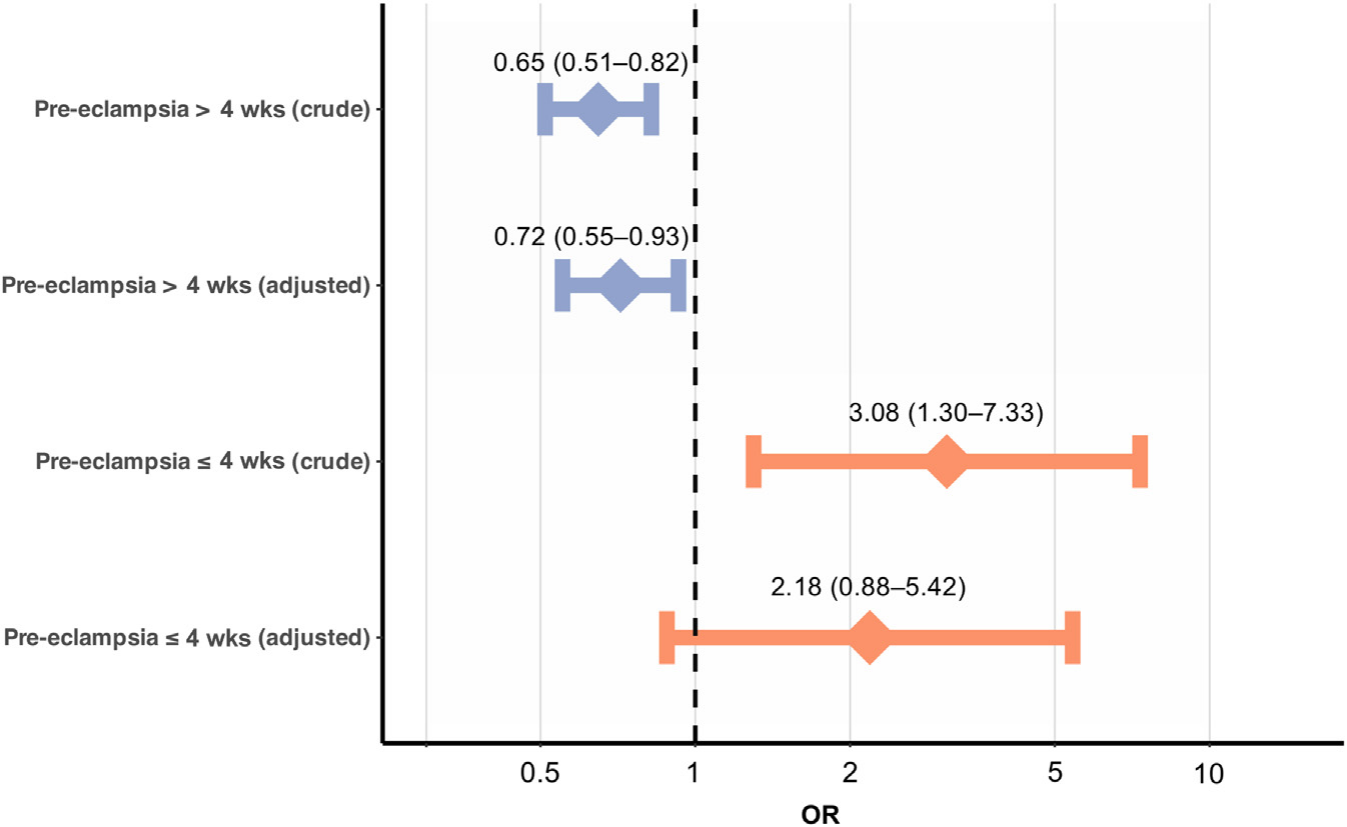
**Association of N-Terminal Pro B-Type Natriuretic Peptide With Pre-eclampsia Development Stratified by Time to Disease Manifestation** ORs are displayed for a one-unit change in log-transformed NT-proBNP values in multinomial logistic regression analyses. Their 95% CIs are given in parentheses. An adjustment was done for maternal age, BMI, nulliparity, systolic blood pressure at sampling, and the sFlt-1/PlGF ratio. BMI = body mass index; NT-proBNP = N-terminal pro B-type natriuretic peptide; sFlt-1/PlGF = soluble Fms-like tyrosine kinase 1/placental growth factor.

To enhance the granularity of the analyses, multinomial logistic regression was performed for pre-eclampsia onset within 4 weeks and after 4 weeks from blood sampling (Figure 4). In the crude model, NT-proBNP was positively associated with the risk of pre-eclampsia within 4 weeks (OR: 3.08; 95% CI: 1.30-7.33; *P* = 0.011), while it was inversely associated with pre-eclampsia beyond 4 weeks (OR = 0.65; 95% CI: 0.51-0.82; *P* < 0.001). The adjusted OR for NT-proBNP and pre-eclampsia within 4 weeks was 2.18 (95% CI: 0.88-5.42; *P* = 0.09), and 0.72 for pre-eclampsia beyond 4 weeks (95% CI: 0.55-0.93; *P* = 0.012).

When pre-eclampsia onset was stratified by gestational age at diagnosis—early-onset (≤34 weeks), late-onset preterm (after 34 weeks but before 37 weeks), and late-onset at term (≥37 weeks)—a similar pattern emerged (Supplemental Figure 1). Higher NT-proBNP levels were associated with an increased risk of early-onset pre-eclampsia, while they were linked to a reduced risk of late-onset pre-eclampsia. The inverse direction of the association between NT-proBNP and late-onset pre-eclampsia was consistent for both preterm and at-term cases.

### Predictive performance of NT-proBNP, sFlt-1/PlGF, and their combination for pre-eclampsia risk

**Figure 5:**
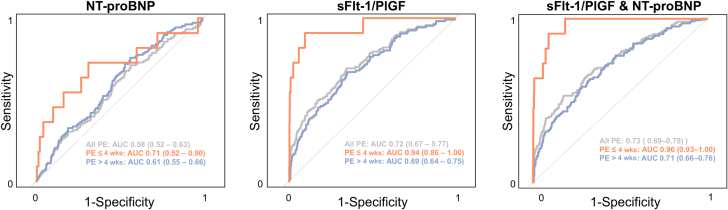
**Predictive Capacity of N-Terminal Pro B-Type Natriuretic Peptide, the Soluble Fms-Like Tyrosine Kinase 1/Placental Growth Factor Ratio, and Their Combination for Pre-eclampsia Prediction** Log-transformed biomarker values were used in receiver operating characteristic analyses to determine the area under the ROC curve (AUC) for predicting any pre-eclampsia (PE), as well as PE onset within and beyond 4 weeks. NT-proBNP = N-terminal pro B-type natriuretic peptide; sFlt-1/PlGF = soluble Fms-like tyrosine kinase 1/placental growth factor.

ROC analyses were performed to evaluate the predictive capacity of NT-proBNP, sFlt-1/PlGF, and their combination for pre-eclampsia risk (Figure 5). NT-proBNP showed poor predictive value overall, with AUCs of 0.58 (95% CI: 0.52-0.63) for all pre-eclampsia cases and 0.61 (95% CI: 0.55-0.66) for pre-eclampsia beyond 4 weeks after sampling. However, its predictive ability was moderate for pre-eclampsia within 4 weeks (AUC: 0.71; 95% CI: 0.52-0.90). In contrast, sFlt-1/PlGF demonstrated stronger predictive power, with an AUC of 0.72 (95% CI: 0.67-0.77) for all cases, 0.69 (95% CI: 0.64-0.75) for pre-eclampsia more than 4 weeks after sampling, and 0.94 (95% CI: 0.86-1.00) for pre-eclampsia within 4 weeks. Combining NT-proBNP with sFlt-1/PlGF provided AUCs of 0.73 (95% CI: 0.69-0.78) for all cases, 0.71 (95% CI: 0.66-0.76) for pre-eclampsia more than 4 weeks after sampling, and 0.96 (95% CI: 0.93-1.00) for pre-eclampsia within 4 weeks, indicating that NT-proBNP did not substantially enhance the predictive performance of sFlt-1/PlGF in any of the groups analyzed.

## Discussion

We identified a nuanced relationship between early third-trimester NT-proBNP levels and pre-eclampsia risk: higher levels were linked to increased risk of pre-eclampsia within 4 weeks, but to reduced risk beyond 4 weeks. However, compared to the sFlt-1/PlGF ratio, NT-proBNP did not enhance the prediction of short- or long-term pre-eclampsia risk (Central Illustration).

**Central Illustration:**
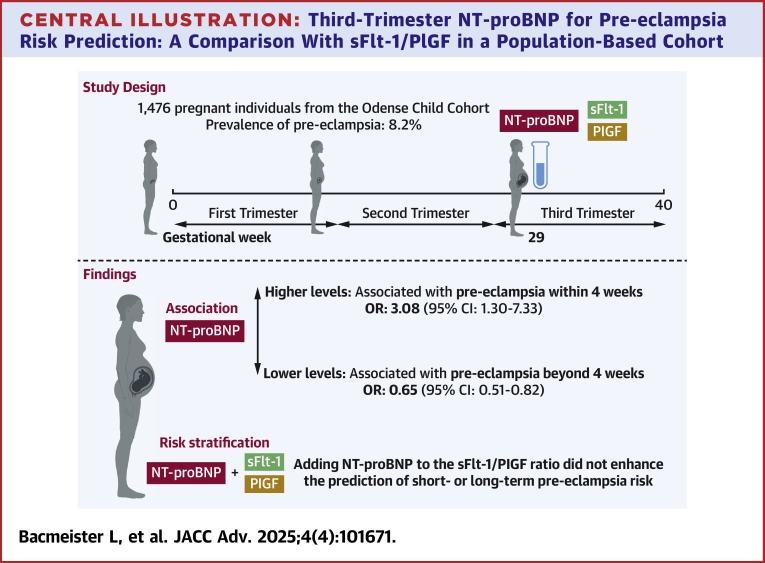
**Third-Trimester NT-proBNP for Pre-eclampsia Risk Prediction: A Comparison With sFlt-1/PlGF in a Population-Based Cohort** Higher natriuretic peptide levels in the early third trimester were linked to pre-eclampsia onset within 4 weeks, while lower levels were associated with onset beyond 4 weeks. However, adding NT-proBNP to the sFlt-1/PlGF ratio did not enhance short- or long-term pre-eclampsia risk prediction in this unselected screening study. ORs and their 95% CIs were obtained by multinomial logistic regression analysis Created in BioRender. Lindner, D. (2025) https://BioRender.com/k11b361. NT-proBNP = N-terminal pro B-type natriuretic peptide; sFlt-1/PlGF = soluble Fms-like tyrosine kinase 1/placental growth factor.

A decrease in vascular resistance during early pregnancy sets the stage for intravascular volume expansion and supports the fetoplacental unit; deviations from this norm are associated with pre-eclampsia.^16^^,^^18^^,^^24^ Reflecting the physiological adaptations during normal pregnancy, natriuretic peptide levels are elevated in the first trimester relative to the nonpregnant state and typically decrease by the third trimester, reflecting stabilization of cardiovascular adaptations.^25^, ^26^, ^27^ NT-proBNP may more reliably reflect cardiovascular alterations during pregnancy, since placental neprilysin degrades BNP, but not its inert byproduct NT-proBNP.^28^^,^^29^

In contrast, earlier studies found higher NT-proBNP levels at the time of pre-eclampsia manifestation.^10^, ^11^, ^12^^,^^30^ Additionally, an inverse correlation between maternal cardiac output and plasma NT-proBNP levels has been reported, alongside a higher prevalence of diastolic dysfunction and cardiac hypertrophy in individuals with pre-eclampsia.^12^^,^^30^^,^^31^ In line with our hypothesis that higher NT-proBNP levels would signal subclinical cardiac dysfunction preceding pre-eclampsia manifesting in temporal proximity, we observed a positive association between NT-proBNP and the risk of pre-eclampsia within 4 weeks. However, NT-proBNP alone had modest predictive capacity for pre-eclampsia within this short-term window (AUC: 0.71), especially when compared to the stronger predictive performance of the sFlt-1/PlGF ratio (AUC: 0.94).

In contrast to our findings in asymptomatic participants of a population-based cohort study, the addition of NT-proBNP to the sFlt-1/PlGF ratio improved the prediction of delivery within 7 days as a result of pre-eclampsia in individuals in whom the disease was clinically suspected.^32^ Hence, NT-proBNP may have value in stratifying prognosis when pre-eclampsia is clinically suspected. As only 2 individuals delivered within 7 days after blood sampling in our study, we were not able to replicate these findings.

As opposed to the associations of natriuretic peptide levels in temporal proximity to pre-eclampsia manifestation, lower first-trimester levels of NT-proBNP have recently been associated with higher risks of pre-eclampsia.^13^ The authors proposed that lower first-trimester NT-proBNP levels might indicate a higher vascular stiffness and a diminished intravascular volume expansion in the first trimester, potentially associated with pre-eclampsia risk.^13^ Our findings of an inverse association between NT-proBNP levels and pre-eclampsia risk beyond 4 weeks corroborate this hypothesis and extend its validity into the early third trimester. Notably, the inverse association with pre-eclampsia risk beyond 4 weeks remained significant after adjustment for confounders, including systolic blood pressure and the sFlt-1/PlGF ratio, and the direction of association did not differ between preterm and at-term manifestations of the disease. However, the predictive capacity of NT-proBNP for pre-eclampsia beyond 4 weeks was very low and did not meaningfully improve that of the sFlt-1/PlGF ratio when combined with it, even though the sFlt-1/PlGF ratio alone also showed limited predictive value. Clinically, this highlights the ongoing challenge of identifying reliable biomarkers for late-onset pre-eclampsia, which is common and causes significant maternal morbidity.

Natriuretic peptides reduce vascular tone, a mechanism that conceptually supports plasma volume expansion essential for the physiological adaptations required for normal pregnancy. Genome-wide association studies have shown that predicted BNP levels are associated with lower blood pressure and a reduced incidence of hypertension in nonpregnant individuals.^33^ Recently, this association was extended to pregnancy, which found that lower genetically predicted BNP levels are linked to an increased risk of hypertensive pregnancy disorders.^34^ Future studies incorporating serial measurements and hemodynamic assessments could help clarify the temporal dynamics of natriuretic peptides across different pre-eclampsia phenotypes and evaluate a potential role of natriuretic peptides as disease-modifiers in pre-eclampsia.

### Strengths and limitations

A major strength of this study is its large, population-based cohort and comprehensive dataset, including angiogenic biomarkers, which enabled an in-depth analysis of the short- and long-term associations between NT-proBNP and pre-eclampsia. However, the observational study design precludes a determination of causality between NT-proBNP levels and pre-eclampsia development. Moreover, the study design does not allow for a direct correlation between NT-proBNP levels and cardiovascular assessments, such as volume status or echocardiographic findings. Assessing first-trimester NT-proBNP levels could have provided further insight into the trajectories of natriuretic peptides throughout pregnancy and their association with pre-eclampsia risk. Future studies should aim to include more ethnically diverse populations, given the higher prevalence of pre-eclampsia in non-White individuals, to enhance generalizability and address disparities in pre-eclampsia research.

## Conclusions

Our findings suggest that unselected NT-proBNP screening during the early third trimester has limited clinical value for short- or long-term prediction of pre-eclampsia. However, future studies focusing on gestational cardiac biomarker levels in individuals who develop hypertensive disorders of pregnancy may be key to identifying predictors of cardiovascular health postpartum.

Perspectives **COMPETENCY IN MEDICAL KNOWLEDGE:** The study's findings highlight a nuanced, time-dependent relationship between natriuretic peptide levels in the early third trimester and pre-eclampsia risk, with higher levels associated with increased risk within 4 weeks and lower levels linked to reduced risk beyond this period. The inverse association observed beyond 4 weeks aligns with prior hypotheses linking lower natriuretic peptide levels to diminished vascular compliance and inadequate plasma volume expansion, both implicated in pre-eclampsia pathophysiology. While prior studies have reported elevated natriuretic peptide levels at pre-eclampsia diagnosis, our finding of a positive association between natriuretic peptide levels and onset of pre-eclampsia in temporal proximity suggests that they may reflect subclinical cardiovascular alterations preceding disease onset.

**TRANSLATIONAL OUTLOOK:** NT-proBNP did not improve predictive performance over the sFlt-1/PlGF ratio, which remains the superior biomarker for short-term pre-eclampsia risk assessment. The modest predictive performance of all biomarkers for pre-eclampsia beyond 4 weeks, however, reinforces the challenge of predicting late-onset pre-eclampsia, which contributes significantly to maternal morbidity. Of note, although NT-proBNP may not enhance population-level screening, it could still play a role in risk stratification when pre-eclampsia is clinically suspected. Future research should incorporate serial NT-proBNP measurements combined with hemodynamic assessments to refine the understanding of natriuretic peptides roles in pregnancy and postpartum cardiovascular health.

## Funding support and author disclosures

Dr Bacmeister is funded by the Berta-Ottenstein-Program for Clinician Scientists, Faculty of Medicine, University of Freiburg and also funded by the Max Grundig Foundation, Baden-Baden, Germany. Dr Birukov is supported by a 10.13039/501100001659German Research Foundation individual fellowship (BI 2427/1-1). Dr Kraeker is funded by the German Center for Cardiovascular Research (FKZ: 81X3100109). Dr Zeller is supported by the German Center for Cardiovascular Research (FKZ: 81Z0710102). Dr Dechend is funded by the 10.13039/501100001659German Research Foundation (DFG 631/15-1, CRC1470, Project-ID 437531118) and the German Center for Cardiovascular Research, Partner Site Berlin, Germany. sFlt-1 and PlGF were initially measured in collaboration with BRAHMS GmbH at their facility in Hennigsdorf, Berlin. For this study, BRAHMS had no further involvement in the collection or interpretation of data, writing of the report, or decision to submit the article for publication. Dr Zeller is listed as a co-inventor of an international patent on the use of a computing device to estimate the probability of myocardial infarction (International Publication No. WO2022043229A1). Dr Zeller is a shareholder in ART.EMIS GmbH Hamburg.
