# Supplementary material for: Third-Trimester NT-proBNP for Pre-eclampsia Risk Prediction: A Comparison With sFlt-1/PlGF in a Population-Based Cohort
Source: JACC Adv. 2025 Mar 19;4(4):101671. doi: 10.1016/j.jacadv.2025.101671 (PMC11968266; doi:10.1016/j.jacadv.2025.101671)
Supplement: Supplemental Data [file mmc1.pdf]

Supplemental Material

Third-Trimester NT-proBNP for Pre-eclampsia Risk Prediction: A Comparison with sFlt-1/PlGF in a Population-Based Cohort

Lucas Bacmeister, MD[a], Annette Buellesbach[a], Dorte Glintborg, MD, PhD[b,c], Jan Stener Jørgensen, MD, PhD[c,d], Birgitte Møller Luef, MD[c,d], Anna Birukov, PhD[e], Adrian Heidenreich, MD[a], Diana Lindner, PhD[a], Till Keller, MD[a], Kristin Kraeker, PhD[f] ,Tanja Zeller, PhD[g,h,i], Ralf Dechend, MD[f,j]*, Marianne Skovsager Andersen, MD, PhD [b,c]*, and Dirk Westermann, MD[a]*

*contributed equally

Address for correspondence:

Lucas Bacmeister, University Heart Center Freiburg – Bad Krozingen, Department of Cardiology & Angiology, University Medical Center Freiburg, Hugstetter Str. 55, 79106 Freiburg, Germany. Mail: lucas.bacmeister@uniklinik-freiburg.de; Twitter: @LucasBacmeister

a) Clinic for Cardiology and Angiology, University Heart Center Freiburg – Bad Krozingen, Medical Center – University of Freiburg, Faculty of Medicine, University of Freiburg, Germany, b) Department of Endocrinology, Odense University Hospital, University of Southern Denmark, Odense, Denmark, c) Institute for Clinical Research, Faculty of Health Sciences, University of Southern Denmark, Odense, Denmark, d) Department of Obstetrics and Fetal Medicine, Odense University Hospital, University of Southern Denmark, Odense, Denmark, e) Department of Nutrition, Harvard T.H. Chan School of Public Health, Boston, MA, f) Experimental and Clinical Research Center, a cooperation between the Max-Delbrück-Center for Molecular Medicine in the Helmholtz Association and the Charité - Universitätsmedizin Berlin, Berlin, Germany, g) Department of Cardiology, University Heart and Vascular Center Hamburg, Medical University Hamburg-Eppendorf, h) Institute for Cardiogenetics, University of Luebeck, University Hospital Schleswig-Holstein i) German Center for Cardiovascular Research, DZHK, partner site Hamburg/Kiel/Lübeck, Hamburg, Germany. j) HELIOS Clinic, Department of Cardiology and Nephrology, Berlin, Germany.

Supplemental Table *S1* Comparison of Characteristics Between Participants Ineligible and Eligible for Study Inclusion

	**Eligible** (N=1476)	**Non-Eligible** (N=685)	**P-value**
Maternal Age (y)	30.0 [27.0,33.0]	30.0 [27.0,33.0]	0.82
BMI (kg/m^2)	23.5 [21.3,26.5]	23.3 [21.1,26.2]	0.092
Pre-eclampsia	121 (8.2%)	52 (7.6%)	0.67
Nulliparous	885 (60.0%)	337 (49.2%)	<0.001
Twin pregnancy	27 (1.8%)	14 (2.0%)	0.74
SBP at first trimester (mmHg)	116 [110,124] *(missing: n=154)*	119 [110,125] *(missing: n=630)*	0.50
Hypertension	58 (3.9%)	20 (2.9%)	0.27
Diabetes	8 (0.5%)	3 (0.4%)	>0.99
Chronic Kidney Disease	4 (0.3%)	0 (0%)	>0.99
Smoking	55 (3.7%)	49 (7.2%)	<0.001
Birth weight (g)	3510 [3160,3850]	3550 [3190,3870]	0.41
Placental weight (g)	610 [530,720]	620 [540,720]	0.29
Female sex	698 (47.3%)	308 (45.0%)	0.43
APGAR at 5 min	10.0 [10.0,10.0]	10.0 [10.0,10.0]	0.28

Baseline characteristics of participants from the Odense Child Cohort who donated blood samples during pregnancy, stratified by eligibility for inclusion in this substudy. Eligibility criteria are outlined in the main body of the manuscript. Continuous variables are presented as medians [Q1, Q3], while binary variables are shown as counts (percentages). P-values were calculated using the Mann-Whitney U test for continuous variables and Fisher's exact test for binary variables. Abbreviations: BMI: Body mass index. SBP: Systolic blood pressure.

Supplemental Table S*2* Baseline characteristics of the study cohort stratified by gestational outcomes

	**No PE**	**Late-onset PE (≥ gw 34)**		**Early-onset PE (< gw 34)**	**P-value**
		At term	*Preterm*		
	(N=1355)	(N=77)	(N=30)	(N=14)	
**Maternal age (y)**	30.0 [27.0,33.0]	30.0 [26.0,34.0]	32.5 [29.0,36.0]	33.5 [26.5,36.0]	0.10
**BMI (kg/m^2)**	23.4 [21.3,26.2]	24.7 [21.7,28.7]	26.9 [22.7,29.0]	27.0 [23.3,31.3]	<0.001
**Nulliparity**	789 (58.2%)	61 (79.2%)	22 (73.3%)	13 (92.9%)	<0.001
**Gestational age at blood sampling (w)**	29.0 [28.4,29.4]	29.0 [28.3,29.4]	29.0 [28.7,29.4]	28.8 [28.5,29.4]	0.54
**Time to pre-eclampsia manifestation (d)**	-	75.0 [64.0,80.0]	45.0 [41.0,50.0]	22.5 [11.0,27.5]	<0.001
**Gestational age at delivery (w)**	40.1 [39.1,41.0]	40.1 [39.7,40.9]	38.1 [37.7,39.1]	37.2 [34.7,38.3]	<0.001
**SBP 1st trimester (mmHg)**	115 [110,122] *(missing: n=139)*	120 [113,130] *(missing: n=7)*	120 [116,131] *(missing: n=7)*	126 [120,131] *(missing: n=1)*	<0.001
**SBP at sampling (mmHg)**	117 [110,125] *(missing: n=54)*	125 [119,130] *(missing: n=2)*	128 [124,138] *(missing: n=1)*	143 [132,148] *(missing: n=1)*	<0.001
**Hypertension**	50 (3.7%)	4 (5.2%)	3 (10.0%)	1 (7.1%)	0.28
**Diabetes**	7 (0.5%)	0 (0%)	1 (3.3%)	0 (0%)	0.18
**Kidney disease**	3 (0.2%)	1 (1.3%)	0 (0%)	0 (0%)	0.35
**Birth weight (g)**	3520 [3180,3860]	3530 [3160,3830]	3030 [2670,3690]	2400 [1900,3110]	<0.001
**Placental weight (g)**	620 [530,720]	600 [530,700]	600 [530,758]	480 [393,573]	0.01
**Female offspring**	640 (47.2%)	35 (45.5%)	16 (53.3%)	7 (50.0%)	0.90
**NT-proBNP (pg/ml)**	47.9 [27.9,79.4]	39.6 [19.7,55.0]	39.7 [21.2,46.7]	78.5 [32.8,143]	0.002
**sFlt-1/PlGF**	3.13 [1.88,5.19]	4.46 [2.77,8.79]	9.63 [5.15,25.5]	34.7 [10.5,148]	<0.001

Baseline characteristics of the study participants stratified by gestational outcomes. At term indicates diagnosis of pre-eclampsia (PE) at gestational week (gw) 37 or later, whereas preterm indicates diagnosis of PE prior to gw 37 (at or later than gw 34).

Continuous variables are reported as medians [Q1, Q3] and binary variables as counts (percentage). P-values were determined using the Kruskal-Wallis test for continuous variables and the chi-squared test for binary variables.

Abbreviations: BMI: Body mass index; SBP: Systolic blood pressure; sFlt-1: sFlt-1/PlGF: soluble fms-like tyrosine kinase-1 to placental growth factor ratio.

Supplemental figures

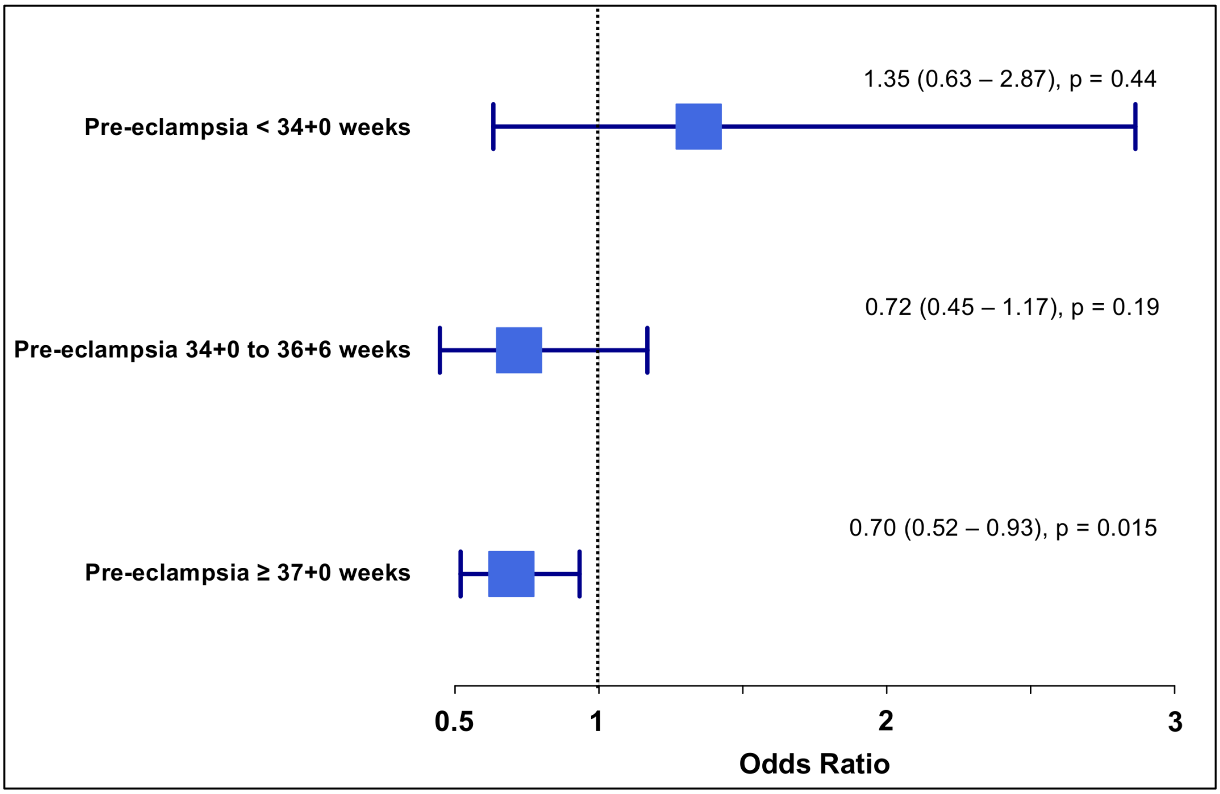

Figure S1: Association of third-trimester NT-proBNP levels and pre-eclampsia risk across gestational ages at diagnosis. Adjusted odds ratios for any pre-eclampsia and for subgroups categorized by gestational age at diagnosis. 95% confidence intervals are indicated in parentheses. Early-onset: <34 weeks; Late-onset preterm: 34-37 weeks; Late-onset at term: >37 weeks. Multinomial logistic regression analyses were adjusted for maternal age, BMI, systolic blood pressure at blood sampling, nulliparity, and the sFlt-1/PlGF ratio.
